# Characterization of Mechanical Stability and Immunological Compatibility for Functionalized Modification Interfaces

**DOI:** 10.1038/s41598-019-43999-6

**Published:** 2019-05-21

**Authors:** Yao-Tsung Hsu, Chih-Yu Wu, Zhen-Yu Guan, Ho-Yi Sun, Chieh Mei, Wen-Chien Chen, Nai-Chen Cheng, Jiashing Yu, Hsien-Yeh Chen

**Affiliations:** 1 Department of Chemical Engineering, National Taiwan University, Taipei, 10617 Taiwan; 2 Department of Orthopedic Surgery, Chang Gung Memorial Hospital, College of Medicine Chang Gung University, Taoyuan, 333 Taiwan; 3 Department of Surgery, National Taiwan University Hospital, Taipei, 10018 Taiwan

**Keywords:** Biomaterials - cells, Biomedical materials

## Abstract

Surface modification layers are performed on the surfaces of biomaterials and have exhibited promise for decoupling original surface properties from bulk materials and enabling customized and advanced functional properties. The physical stability and the biological compatibility of these modified layers are equally important to ensure minimized delamination, debris, leaching of molecules, and other problems that are related to the failure of the modification layers and thus can provide a long-term success for the uses of these modified layers. A proven surface modification tool of the functionalized poly-para-xylylene (PPX) system was used as an example, and in addition to the demonstration of their chemical conjugation capabilities and the functional properties that have been well-documented, in the present report, we additionally devised the characterization protocols to examine stability properties, including thermostability and adhesive strength, as well as the biocompatibility, including cell viability and the immunological responses, for the modified PPX layers. The results suggested a durable coating stability for PPXs and firmly attached biomolecules under these stability and compatibility tests. The durable and stable modification layers accompanied by the native properties of the PPXs showed high cell viability against fibroblast cells and macrophages (MΦs), and the resulting immunological activities created by the MΦs exhibited excellent compatibility with non-activated immunological responses and no indication of inflammation.

## Introduction

Functionalization of the surfaces of biomaterials by performing surface modification has recently drawn increasing attention and has shown itself to be a promising means to flexibly decouple surface properties from bulk materials to enable customized and precisely controlled interfacial environments to accomplish tasks that the original material property cannot^1–3^. For instance, functional groups, including amines, alcohols, aldehydes, anhydrides, activated carboxylic acids, activated esters, maleimides, and alkynes, are chemically installed onto the surface of materials that can further provide specific conjugation linkage toward more delicate molecules of interest^4,5^, or by performing physical strategies allow non-specific binding of molecules to provide the desired properties^6–8^. These chemical and physical approaches have been discussed for the modification of a wide range of substrate materials^9–12^ and have been demonstrated successfully with functionalized molecules, such as biotins, proteins, saccharides, DNA, hydrogels, polymers, enzymes, and cells^13–15^, and for targeted applications in anti-fouling modifications to suppress nonspecific protein binding or bacteria^16–19^, controlling immobilization of cell-adhering peptides to manipulate cellular activities of stem cells and somatic cells^3,20,21^, and defining coupling of antibodies and proteins^22–24^. In addition, the creation of the balance in both the specific functionality and the spatial recognition with respect to 3D cellular responses, substrates/devices with curvature and the complex geometry were also demonstrated with promising results^25,26^.

With the wide applicability and the emerging utilization of the modifications for functionalized biomaterials in the applications where sophisticated biological conditions are involved (such as for tissue engineering materials, implantable devices, and injectable carriers)^27–29^, a more stringent requirement of the modification stability and the immunological compatibility has become a critical topic, especially for the modified materials/devices in long-term use. A durable modification coating is crucial to ensure the absence of an unacceptable deformation^30^ and/or poor adhesion property-caused debris to prevent the coating failures that may potentially result in a high risk of toxicity to the surrounding tissue^31^. In addition, firmly attached biomolecules allow defined biological structures to be maintained over extended periods of time^32^, avoid the leaching of the toxic or overdose substances into the surrounding cells and tissues, and enable a confined and targeted treatment efficacy in a local region. Moreover, the compatibility of the modification coating is closely associated with the durability of the modified layers, and poor cellular viability and/or immunological responses can be triggered by an unstable modification layer and toxic modification procedure/composition^33^. However, compared to the majority of functional capability demonstrations, the post-modification properties of stability/immunological compatibility have only been discussed sporadically or prematurely in the context of the protein adsorption, the cell viability, and the cell attachment behavior^34–36^. In the pilot study, we therefore present a detailed and systematic investigation of the stability/compatibility by using a vapor-phased modification of poly-*para*-xylylenes (PPXs) as a demonstration and intend to establish a standard for early assessment of the modification stability and compatibility before entering a more critical and complicated animal study or clinical trial. The functionalized PPXs are synthesized via chemical vapor deposition (CVD) polymerization from substituted [2,2] paracyclophanes^37^, and have been proven to be a powerful interlayer material for the modification of a variety of substrate materials^19,38–40^, and for the manipulation of biointerfaces. The PPX coatings represent excellent candidates for prospective interface designs and require the investigation of the biocompatibility with respect to the coating durability and the immunological compatibility. The mechanical adhesive property and the thermostability of the PPX-modified surfaces were examined, and more importantly, in light of the role played by macrophages (MΦs) in the host response to modified surfaces, the measurements of the MΦ viability, the proliferation, morphological changes, the production of the pro-inflammatory cytokine tumor necrosis factor-alpha (TNF-α) interleukin 1-beta (IL-1β), and levels of the immune-modulator nitric oxide (NO) have been analyzed for the modified functional surfaces^41,42^. Taken together, the results of this study confirm that the uncompromised immunological activity elicits a minimal inflammatory response likely to result in both improved local wound healing and fewer systemic complications. These results should provide essential insights into advanced biointerface design using functional PPX coatings.

## Results and Discussion

### Stability of the surface modification

A photoreactive poly-*para*-xylylene, poly(4-benzoyl-*p*-xylylene-*co*-*p*-xylylene) (hereafter referred to as benzoyl-PPX) and a maleimide-functionalized poly-*para*-xylylene, poly(4-N-maleimidomethyl-*p*-xylylene-*co*-*p*-xylylene) (hereafter referred to as maleimide-PPX) were selected for the surface modification demonstration and were first synthesized by CVD polymerization. An *in situ* installed quartz crystal microbalance (QCM) was used to characterize the coating thickness during the CVD process, and the control of the film growth rate was maintained at approximately 0.5 Å/s to ensure the quality of the coating. The resultant thickness of the coating was approximately 2000 Å with a uniformity of ±70 Å. The modification of benzoyl-PPX coating provides a reactivity analogous to the benzophenone moiety that can rapidly conjugate with molecules via benzophenone triplet insertion into *C-H* or *N-H* bonds under UV irradiation^43,44^. However, the maleimide-PPX modification provides the accessibility to Michael-type nucleophilic addition between the maleimide group and a target molecule that contains a thiol group^45^. Three functional molecules, chlorhexidine (CHX, a commonly used antibacterial agent)^46^, bone morphogenetic protein 2 (BMP-2, an osteoinductive growth factor)^47,48^, and Arg-Gly-Asp-Ala-Cys-Cys (RGDACC, a cell adhesive peptide)^49^ were selected for the immobilization of the PPX coatings, as they have been demonstrated to create biointerfaces with diverse functions, including antibacterial activity^19^, regulated osteogenesis^23^, and enhanced cell attachment^50^, respectively. The immobilization exploited the aforementioned conjugations provided by benzoyl-PPX or maleimide-PPX; subsequently, these PPXs coatings were modified on gold-coated silicon substrates. More specifically, CHX was immobilized on the benzoyl-PPX surface via photochemical reaction, and the BMP-2 and RGDACC were immobilized on the maleimide-PPX surface via the maleimide-thiol chemistry (Fig. S1).

**Figure 1:**
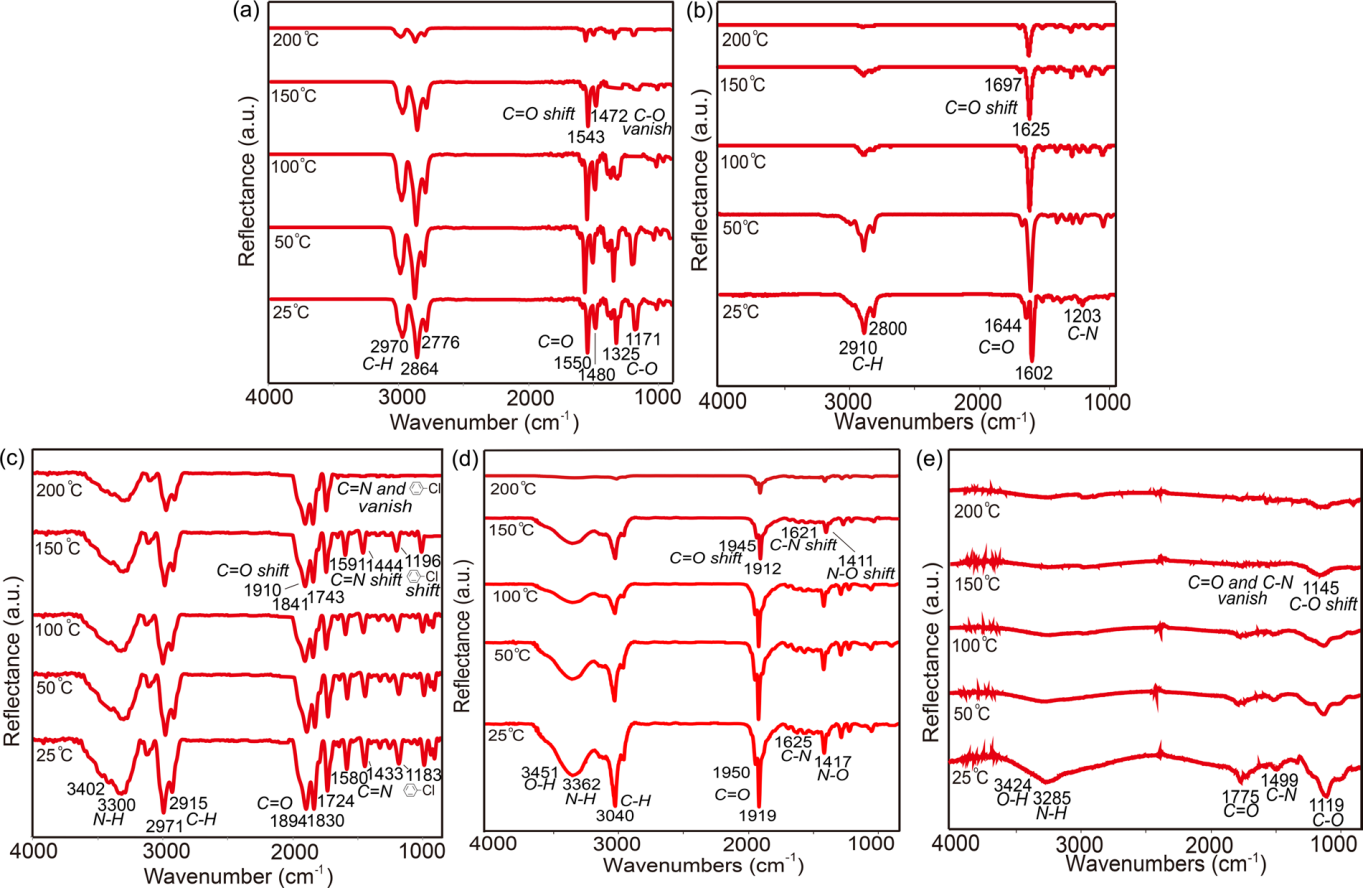
Characterization of the thermostability using IRRAS for the modified surfaces on (**a**) bare benzoyl-PPX, (**b**) bare maleimide-PPX, (**c**) CHX-immobilized surface, (**d**) BMP-2-immobilized surface, and (**e**) RGDACC-immobilized surface. The spectra were recorded on the same samples for the selected surfaces with elevated temperatures from 25 °C to 200 °C.

The resulting thermal stability of the modified surfaces/biointerfaces including the bare PPX coatings (benzoyl-PPX or maleimide-PPX) and the three activated functional surfaces (CHX, BMP-2, and RGDACC) were characterized using infrared reflection absorption spectroscopy (IRRAS) analysis. As indicated in Fig. 1, the characterizations were performed first at 25 °C, and the characteristic bands of *C-O* (1171 cm^−1^ and 1325 cm^−1^), *C*=*O* (1480 cm^−1^ and 1550 cm^−1^) and *C-H* (2776 cm^−1^, 2864 cm^−1^ and 2970 cm^−1^) were detected for the bare coating of benzoyl-PPX. Similarly, peaks of *C-N* (1203 cm^−1^), *C*=*O* (1602 cm^−1^ and 1644 cm^−1^) and *C-H* (2800 cm^−1^ and 2910 cm^−1^) from the maleimide group were detected in the bare maleimide-PPX coating. In addition, conjugation using the benzoyl-PPX coating with chlorhexidine (CHX) were showing a chlorophenyl peak at 1183 cm^−1^, *C*=*N* peaks at 1433 cm^−1^ and 1580 cm^−1^, *C*=*O* peaks at 1724 cm^−1^, 1830 cm^−1^ and 1894 cm^−1^, *C-H* peaks at 2915 cm^−1^ and 2971 cm^−1^ and *N-H* peaks at 3300 cm^−1^ and 3402 cm^−1^. The conjugations using maleimide coating to immobilize BMP-2 and RGDACC were, however, showing representative peaks at 1417 cm^−1^ (*N-O*), 1625 cm^−1^ (*C-N*), 1919 cm^−1^ and 1950 cm^−1^ (*C*=*O*), 3040 cm^−1^ (*C-H*), 3451 cm^−1^ (*O-H*) and 3362 cm^−1^ (*N-H*) for the BMP-2, and peaks at 1119 cm^−1^ (*C-O*), 1499 cm^−1^ (*C-N*), 1775 cm^−1^ (*C*=*O*), 3285 cm^−1^ (*N-H*) and 3424 cm^−1^ (*O-H*) for the RGDACC. The data above not only verified the successful conjugation of the immobilized molecules but also showed the stable results of these coatings/modifications under the tested thermocondition (25 °C). The thermostability test continued by exposing the same samples under elevated temperatures from 25 °C to 50 °C, 100 °C, 150 °C and, finally, 200 °C. As indicated in the IRRAS data, a decrease in the stability started to be revealed under the thermocondition above 150 °C by showing decreased absorption peaks. More specifically, the *C*=*O* peak decreased significantly under the thermocondition above 150 °C and shifted from 1480 cm^−1^ and 1550 cm^−1^ to 1472 cm^−1^ and 1543 cm^−1^, respectively, for the bare benzoyl-PPX coating. The *C-O* peak even vanished under 150 °C. For the bare maleimide-PPX coating, the *C*=*O* peak decreased under 150 °C, and shifted peaks were found for 1602 cm^−1^ and 1644 cm^−1^ to 1625 cm^−1^ and 1697 cm^−1^, respectively. Furthermore, similar peak shift or vanishing was also detected for the molecule-immobilized surfaces of the CHX-immobilized surface (chlorophenyl peak shifted from 1183 cm^−1^ to 1196 cm^−1^, the *C*=*N* peaks shifted from 1433 cm^−1^ and 1580 cm^−1^ to 1444 cm^−1^ to 1591 cm^−1^, *C*=*O* peaks shifted from 1724 cm^−1^, 1830 cm^−1^ and 1894 cm^−1^ to 1743 cm^−1^, 1841 cm^−1^ and 1910 cm^−1^). The BMP-2-immobilized surface (vanished peak for chlorophenyl peak and *C*=*N* peaks under 200 °C, decreased and shifted peaks for the *N-O*, *C-N* and *C*=*O* from 1417 cm^−1^ to 1411 cm^−1^, 1625 cm^−1^ to 1621 cm^−1^, 1919 cm^−1^ to 1912 cm^−1^ and 1950 cm^−1^ to 1945 cm^−1^, respectively); RGDYCC-immobilized surface (vanished peaks for *C-N* and *C*=*O* above 150 °C, decreased and shifted peak for *C-O* from 1119 cm^−1^ to 1145 cm^−1^ above 150 °C). Nevertheless, for most circumstances of biological applications that favor a processing temperature of room temperature (approximately 25 °C), the coatings and the modified layers have provided stable and sustained properties for the uses under the conditions (below 150 °C).

**Figure 2:**
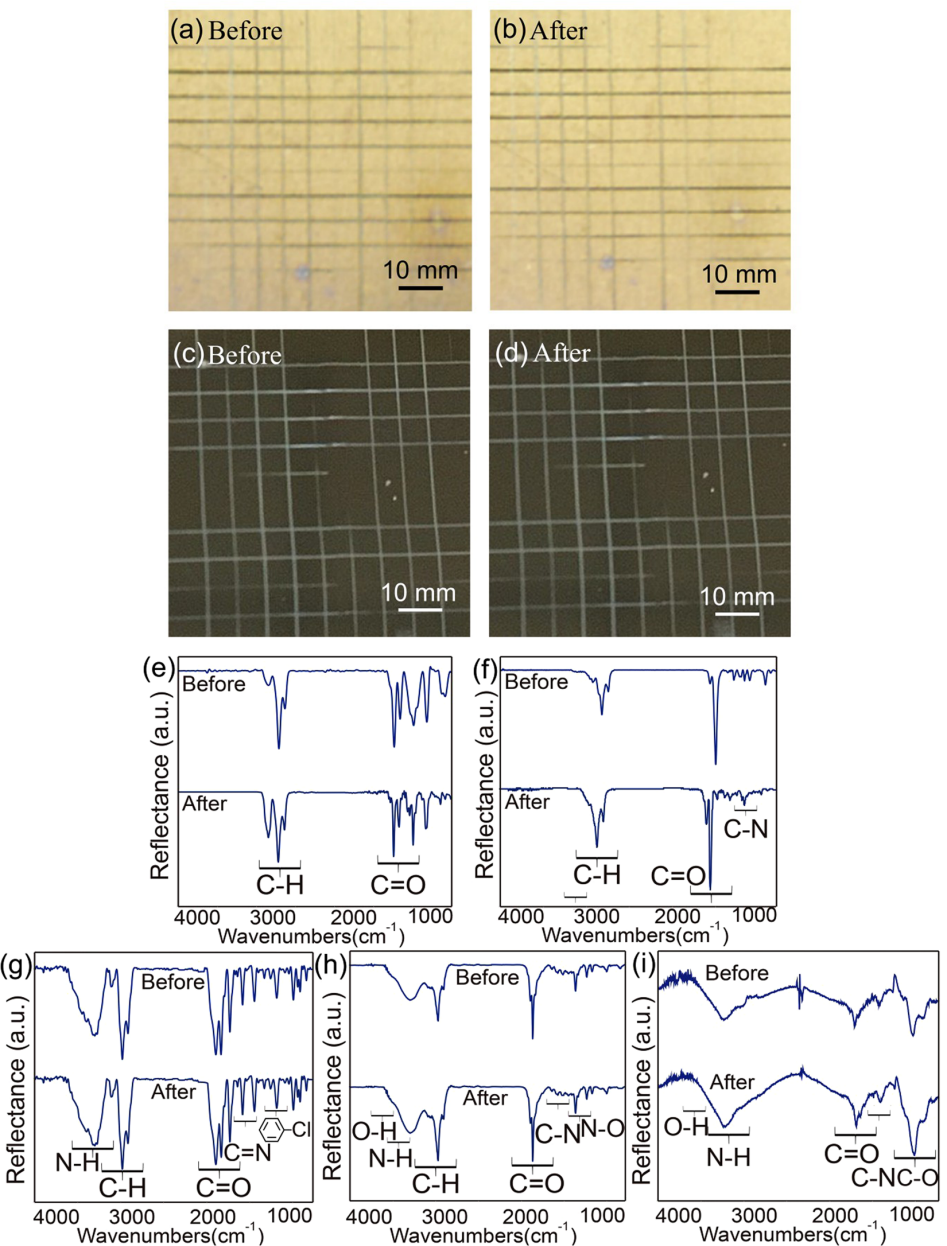
Characterization of the mechanical stability by using the cross-cut tape adhesion test and IRRAS for the modified surfaces. (**a**) Images from the digital camera of bare PPX coating modified on a gold substrate before and after the cross-cut tape adhesion test. The bare coating of benzoyl-PPX was used for the demonstration. (**b**) Images from the digital camera of the molecule immobilization modification on a silicon substrate before and after the cross-cut tape adhesion test. The molecule of BMP-2 was used for the demonstration. IRRAS spectra were also recorded before and after the cross-cut tape adhesion test for the modified surface on (**e**) bare benzoyl-PPX, (**f**) bare maleimide-PPX, (**g**) CHX-immobilized surface, (**h**) BMP-2-immobilized surface, and (**i**) RGDACC-immobilized surface.

The adhesive stability of the coatings and the modification layers against the substrates was further examined using a cross-cut tape adhesion test. A multiblade cross-cut tester was exploited to create two sets of crosswise scratches on the modified substrate, and a piece of Scotch tape was later applied to the samples within approximately 0.5 to 1.0 second followed by a tape-removal process with an angle of 60 degrees for the pulling direction with a steady pull. The tested samples were analyzed using a digital camera to obtain images of the samples before and after the tape was removed. No discernible damage or delamination was found at the cut edges or on the area of the remaining modified layers (Fig. 2a–d). In addition, the adhesive stability with respect to the change in chemical composition was further characterized using IRRAS analysis. As shown in Fig. 2e–i, the resulting IRRAS spectra indicated consistency for all of the samples tested, including bare coatings of benzoyl-PPX and maleimide-PPX, as well as the modifications of immobilized CHX, BMP-2, and RGDACC surfaces, and no discernible changes in the intensity of characteristic absorption bands and/or band position shifts were detected. Finally, the concern of a potential leaching problem was conducted by soaking the samples in a buffer solution at 37 °C for 30 days, and no trace of detectable molecules (CHX, BMP-2, RGDACC) was found, verifying the stable binding of these molecules, and a firmly adhered coating/modification layer was unambiguously confirmed. The results above collectively confirmed: (i) the availability of the functional moieties/molecules of the coating and modification layers; (ii) the stability of these functional moieties/molecules under 150 °C (and these functional moieties/molecules are sufficiently useful for vast biointerface applications at room temperature); and (iii) robust adhesive strength of the coating/modification layers that are durable to avoid delamination and/or leaching concerns.

### Cell viability

**Figure 3:**
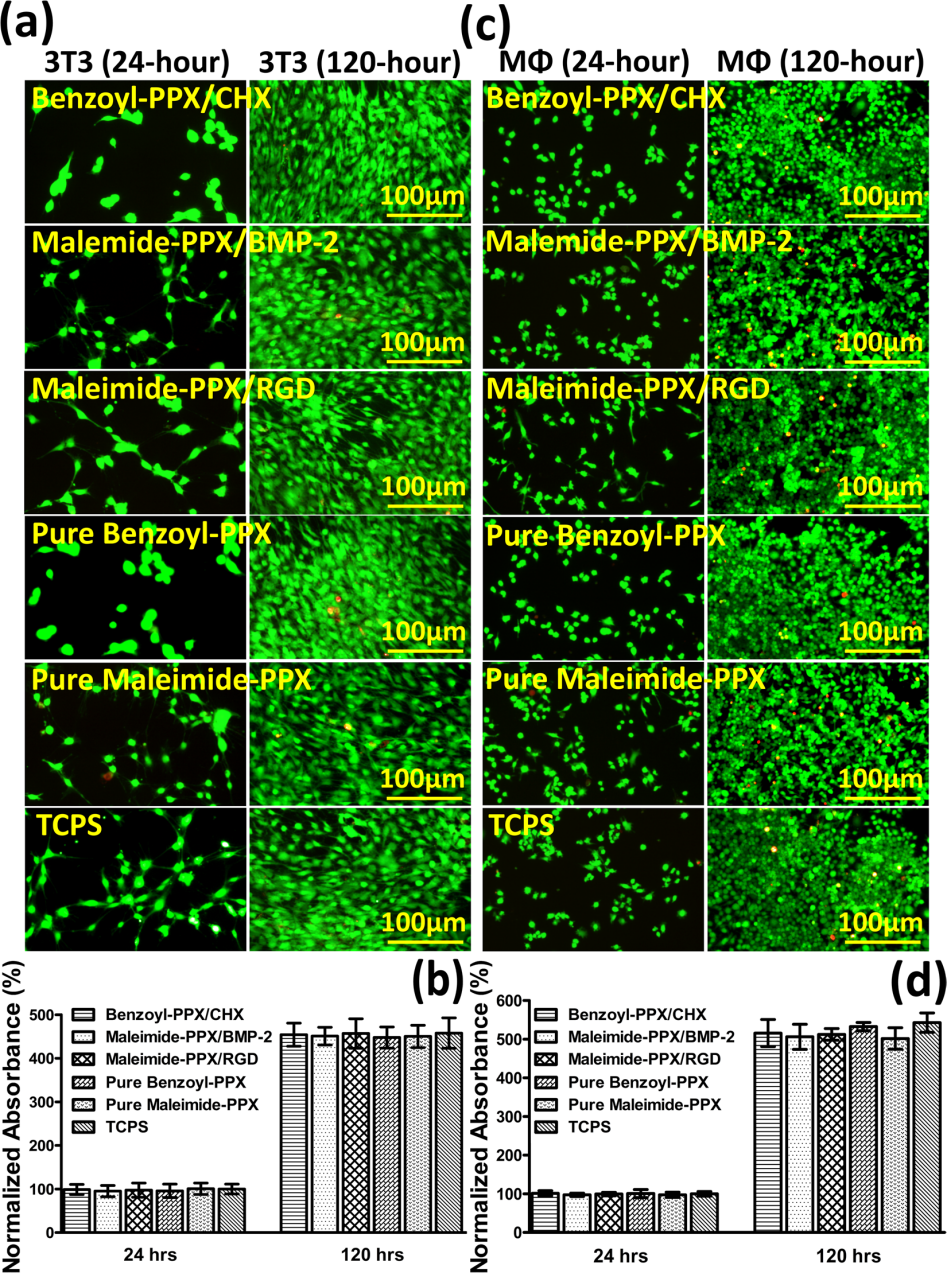
Analysis of cell viability and proliferation for the modified surfaces including the bare coatings of benzoyl-PPX and maleimide-PPX, and the modification of immobilized CHX, BMP-2, and RGDACC surfaces. (**a**) Fluorescence micrographs of the cultured 3T3 fibroblasts on the studied surfaces after 24 and 120 hours. (**b**) Statistical analysis of the 3T3 fibroblasts viability by the MTT assay on the surfaces after 24 and 120 hours. (**c**) Fluorescence micrographs of the cultured MΦs on the studied surfaces after 24 and 120 hours. (**d**) Statistical analysis of the MΦs viability by the MTT assay on the surfaces after 24 and 120 hours. Each bar in (**b**,**d**) represented the mean values (±SD) of three independent experiments.

The important question of the cell compatibility and cytotoxicity of the coating/modification layers was investigated, and the viability of cultured 3T3 fibroblasts was examined on the modified surfaces. Short-term (24 hours) and long-term (120 hours) viability was examined by allowing 3T3 fibroblasts to grow on the same modified surfaces including bare coatings of benzoyl-PPX and maleimide-PPX, as well as the modification of immobilized CHX, BMP-2, and RGDACC surfaces. The tested surfaces were also compared in parallel to a nontoxic control surface on a commercial tissue culture polystyrene (TCPS). The cell proliferation and the viability were assessed in two ways. First, a two-component kit was employed with ethidium bromide and calcein AM to assess the percentage of live/dead cells on the test surfaces. The dead cells with damaged membranes let in the ethidium bromide which stained the cells red, whereas the live cells blocked the ethidium and were able to convert the calcein AM to fluorescent calcein, which stained the cells green (Fig. 3a). Notably, the number of dead cells was found to be insignificant (less than 0.5%, negligible sight of red signals) compared to the living cells (green signals), and the results were consistent for all tested surfaces. With a positive anticipation, these cells were all grown healthily toward a longer term of 120 hours incubation on the studied surfaces with the same low level of dead cells. Moreover, the metabolic activity of the cell was measured using a colorimetric method with 3-(4,5-dimethylthiazol-2-yl)-2,5-diphenyltetrazolium bromide (MTT) reagent. As shown in Fig. 3b, the MTT assay data showed viable 3T3 cells growing on all the studied surfaces, and the 3T3 cells were equally viable on the TCPS surface. More specifically, normalized values (with respect to the value on the TCPS surface) of cell viability were determined for bare benzoyl-PPX coating (95.8 ± 15.3%), bare maleimide-PPX coating (100.7 ± 13.2%), CHX-immobilized (98.8 ± 11.7%), BMP-2-immobilized (95.2 ± 12.9%), and RGDACC-immobilized (97.6 ± 6.4%). Furthermore, the assessment of long-term compatibility evaluated at 120 hours was showing a profound increase in MTT signal (as compared to the results from 24 hours), and the values were detected and calculated accordingly for TCPS (457.9 ± 34.7%), bare benzoyl-PPX coating (447.6 ± 24.4%), bare maleimide-PPX coating (450.2 ± 25.6%), CHX-immobilized (454.2 ± 26.6%), BMP-2-immobilized (450.8 ± 20.2%), and RGDACC-immobilized (456.9 ± 33.8%), with similar consistency. Additional images showing a confluent cell growing pattern and cell morphology on the studied surfaces were also observed, and the data are included in the supporting information in Fig. S2. The cell viability results also showed implications that the surface modifications of using PPX coatings and the further immobilizations are highly compatible with minimalized influence on the initial cell attachment as well as showing negligible toxicity for cell growth and proliferation. The results have also unambiguously supported the abovementioned stability study that the excellent adhesive property of the coatings/modifications can prevent failures from debris and/or leaching molecules to result in a risk of the toxicity to the surrounding cells and tissues.

### Immunological compatibility

**Figure 4:**
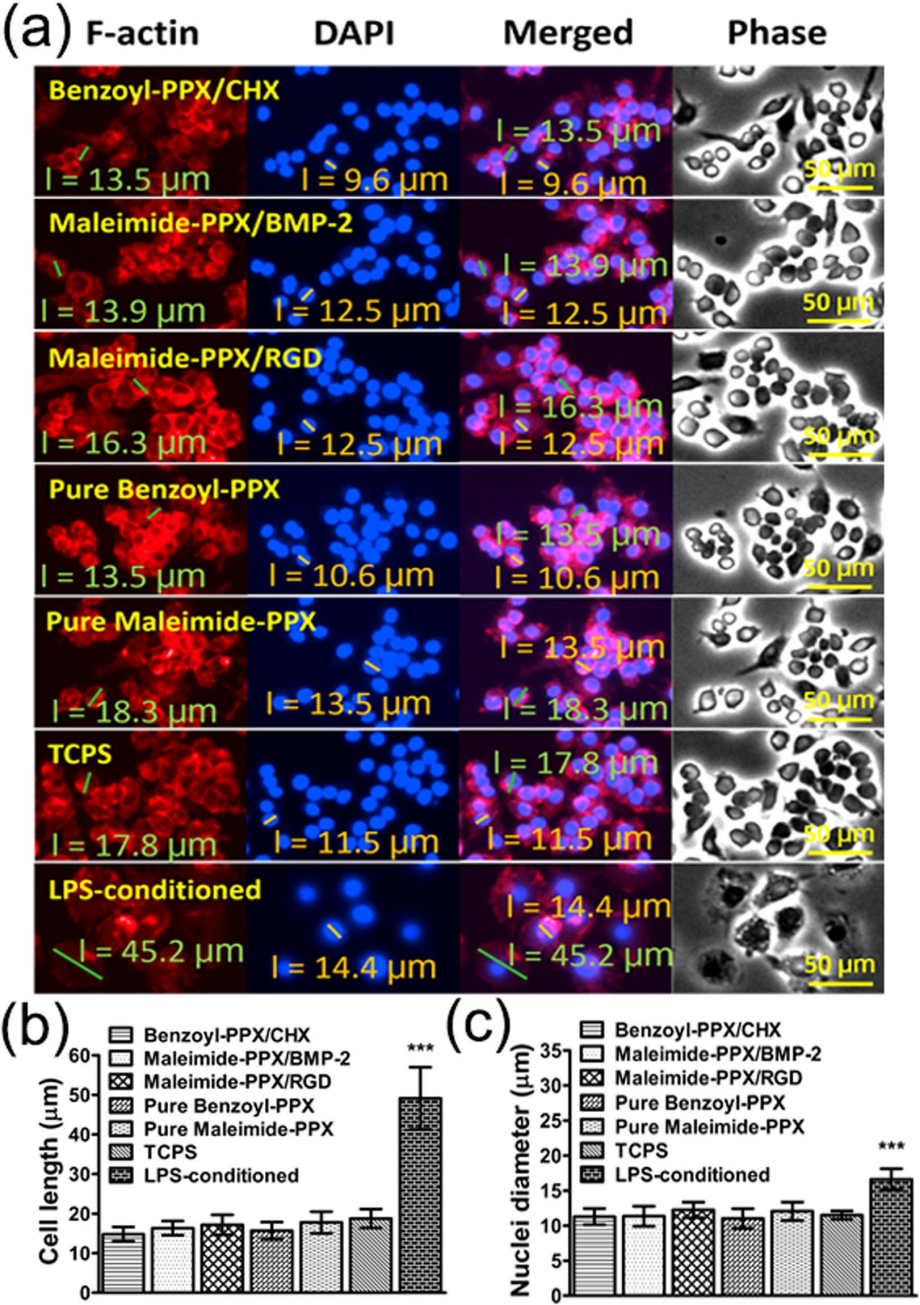
Analysis of the MΦ morphology on the modified surfaces. (**a**) Compiled images including the fluorescence micrographs of the stained cytoskeleton (F-actin, red channel) and nuclei (DAPI, blue channel), and the overlaid images of both red and blue channels. Phase-contrast micrographs were also shown for the comparison. The statistical results of (**b**) cell length and (**c**) nuclei diameter of the cultured MΦs on the studied surfaces. Data analysis was performed after 24 hours of cell culture (***P < 0.001).

Important compatibility factors regarding the immunological responses of short- and long-term macrophage MΦ activity on the modified surfaces were examined. Assessments including the live/dead assay and the MTT cell viability at 24 and 120 hours, similar to the tests examined for the 3T3 fibroblast, were first conducted for the MΦs, and consistent results in Fig. 3c,d of negligible dead cells and high values of cell viability and proliferation were, as anticipated, discovered for the cultured MΦs on the studied surfaces, including bare coatings of benzoyl-PPX and maleimide-PPX, and the modifications of immobilized CHX, BMP-2, and RGDACC surfaces, as well as the control surface of a TCPS. Statistically, the normalized MTT data at 24 hours were showing viability of MΦs for bare benzoyl-PPX coating (100.7 ± 10.3%), bare maleimide-PPX coating (97.5 ± 7.0%), CHX-immobilized (101.5 ± 6.6%), BMP-2-immobilized (97.3 ± 4.2%), and RGDACC-immobilized (99.4 ± 4.6%) compared to the TCPS surface. For the proliferated MΦs in 120 hours, increased MTT values were shown for TCPS (542.9 ± 25.2%), bare benzoyl-PPX coating (532.6 ± 10.7%), bare maleimide-PPX coating (501.8 ± 27.9%), CHX-immobilized (515.8 ± 35.0%), BMP-2-immobilized (506.2 ± 32.3%), and RGDACC-immobilized (512.5 ± 15.2%), with consistency. Compared to a classically activated and inflammatory MΦ that presents a decreased rate of proliferation^51^, the results indicated the modified surface can support the MΦ proliferation with high values of viability, and without eliciting the activation of MΦs. The morphology of MΦ, a strong indicator of the activation^52^, was also analyzed for the modified surfaces. As indicated in Fig. 4a, the images from the phase-contrast and the fluorescence microscopy showed a typical oval-shaped MΦ with less stretched cell morphology, and the statistical measurements for the diameters of the cells and corresponding nuclei were approximately 13.5 (±8.6%) µm and 9.6 (±33.3%) µm, respectively, for CHX-immobilized surface, 13.9 (±72.7%) µm and 12.5 (±26.7%) µm (BMP-2-immobilized surface), 16.3 (±25.1%) µm and 12.5 (±9.5%) µm (RGDACC-immobilized surface), 13.5 (±21.4%) µm and 10.6 (±16.1%) µm (bare benzoyl-PPX), 18.3 (±16.7%) µm and 13.5 (±12.1%) µm (bare maleimide-PPX), and 17.8 (±18.9%) µm and 11.5 (±13.8%) µm (TCPS surfaces), compared to the activated MΦ phenotypes which were reported to exhibit an altered cell shape of elongated overall cell length and nucleus^53,54^. The statistical results revealed no significant alteration and elongation of the MΦs on the modified surfaces (Fig. 4b,c). An additional experiment was performed to purposely activate the MΦs in a lipopolysaccharide (LPS)-conditioned environment, and a noticeable increase in the cell length due to the expansion of the cytoplasm (45.2 (±18.2%) µm) as well as the expanded nucleus (14.4 (±26.7%) µm) were observed. Statistically, the activation (LPS-conditioned MΦs) caused an increase of 261.9 ± 42.0% in the cell size and 144.3 ± 13.2% in the nuclear diameter when comparing to the non-activated MΦs on the studied surfaces. Similar analysis was also performed for 120 hours of cell culture and was showing consistent results, and the data are included in the supporting information in Fig. S3.

**Figure 5:**
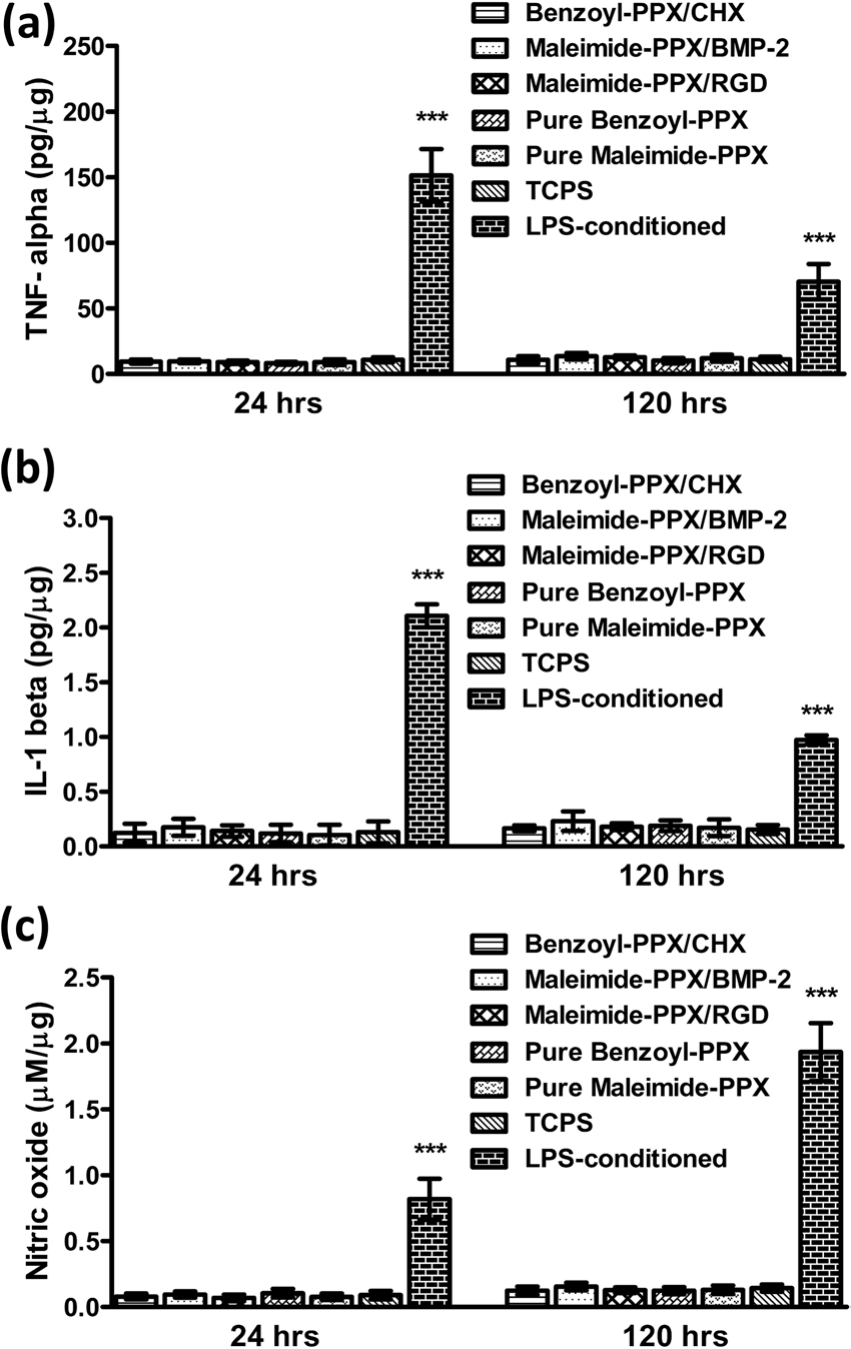
Characterization of the inflammatory responses of the cultured MΦs on the modified surfaces. Statistical analysis of the secreted inflammatory indicators including (**a**) TNF-α, (**b**) IL-1β, and (**c**) NO, from the cultured MΦs on the studied surfaces. Analysis was performed after 24 hours and 120 hours of cell culture. Each bar represents the mean values (±SD) of three independent experiments (***P < 0.001). The results were normalized with respect to the amount of intracellular total proteins.

Finally, the inflammatory responses, such as the pro-inflammatory cytokines and the signaling factors released by the MΦs, including TNF-α, IL-1β, and nitric oxide (NO), were analyzed for MΦs on the studied surfaces. The cytokines of TNF-α and IL-1β are reported being the characteristic signaling molecules involved in the response of MΦs to foreign materials, and the upregulation of these cytokines is an accurate measure indicative of inflammation^42^. However, the secretory product of NO from mammalian cells is another indicator characterizing the initiations of host defense, homeostatic and developmental functions by the intercellular signaling of MΦs, and the activation of the inflammatory process corresponds to an increase of NO production^41^. The expressed signals of TNF-α, IL-1β, and NO on the studied surfaces including the bare coatings of benzoyl-PPX and maleimide-PPX, and the modifications of immobilized CHX, BMP-2, and RGDACC surfaces, were recorded in parallel with the control surface of a TCPS and the activated LPS-conditioned surface, and the results were statistically analyzed and compared, as shown in Fig. 5. Compared to the high degree of expressed signals detected for the LPS-conditioned surface which was indicative of an induced inflammatory sample, the signals, by contrast, were showing lowered levels of expression on the studied surfaces, and such low expression was also found comparable to the expression on the control surface of TCPS with the same low level of the signal. The results have verified a non-inflammatory state for MΦs on the modified surfaces, and the results were valid for the cultured surfaces at both 24 hours and 120 hours.

## Conclusions

Modification layers that are comprised of durable and stable properties, as well as the accounts of compatibility to minimize toxicity and the resistance to inflammatory responses, can ensure a long-term success for the uses in biomaterials and biotechnological applications. There properties were studied and are suggested, as well as the functional demonstrations for the modified layers, as an early validation protocol that is critical for the determination of continuation to enter a time- and cost-consuming process of animal study and/or clinical trial. This study additionally demonstrated the missing information of the stability and the immunological compatibility for the functionalized PPX coatings and have indeed provided the supports to confirm them being a robust tool to fulfill the critical needs for biointerface engineering. The results should help to inform the users and investigators in conducting biomaterial modifications, as well as benefiting the related industrial products.

## Experimental Section

### Surface modifications

A benzoyl-PPX coating was synthesized from 4-benzoyl-[2.2]paracyclophane via CVD polymerization process based on reported conditions^43^. A sublimation temperature of approximately 120 °C was used to sublime 4-benzoyl-[2.2]paracyclophane and the pyrolysis zone was approximately 800 °C to cleave the C-C bonds of the paracyclophanes to generate the corresponding *p*-quinodimethanes. QCM analysis (STM-100/MF, Sycon Instruments) was installed in the deposition chamber and used to characterize the coating thickness during the coating process. A controlled growth rate of approximately 0.5 Å/s under 75 mTorr, and the rate of carrier argon was approximately 30 sccm. For a maleimide-PPX coating, 4-N-maleimidomethyl-[2.2]paracyclophane was synthesized first according to the literature^55^. 4-N-Maleimidomethyl-[2.2]paracyclophane was then sublimated in the sublimation zone at approximately 120 °C. The pyrolysis zone was maintained at 560 °C to cleave the *C-C* bond. Deposition rates were maintained at approximately 0.5 Å/s under 75 mTorr that was monitored via QCM analysis. The rate of carrier argon was approximately 50 sccm.

### Immobilization

CHX (Sigma-Aldrich), BMP-2 (R&D Systems) and RGD (Yao-Hong Biotechnology Inc.) were obtained commercially and were reconstituted in a stock solution at −20 °C. For CHX immobilization, the photoimmobilization experiment was conducted by exposing samples to UV light (Univex) with a 365 nm wavelength and a maximum intensity of 65 mW/cm^2^ for 5 minutes. The samples were rinsed thoroughly with deionized water and dried by a stream of compressed nitrogen gas. However, immobilization of BMP-2 and RGD via a maleimide-thiol coupling reaction was performed by allowing the maleimide-PPX-modified substrates to react with the sample solutions at 4 °C and pH 6.5–7.5 for 6 hours. Dithiothreitol (5 mM, Sigma-Aldrich) was used as a reducing agent. A rinsing process was performed three times with phosphate-buffered saline (PBS) to remove any unbound molecules.

### Stability test of the modification layer

Adhesive strength of the modification layers was examined by using a multi-blade cross-cut tester (ZCC 2087 cross-cut tester, Zehntner) on the modified surfaces including bare coatings of benzoyl-PPX and maleimide-PPX, as well as the modifications of immobilized CHX, BMP-2, and RGDACC. In detail, the cross-cut tester was utilized to create two sets of crosswise scratches on the studied surfaces. Subsequently, a piece of Scotch tape was applied and removed steadily from the scratched samples within 0.5 to 1.0 second and with a pulling direction angle of 60 degree. The resulting samples were imaged with a digital camera (Nikon, D80 with lens 18–200 mm). The stability of the chemical composition of the modification layers and the attached molecules was verified by using IRRAS (Spectrum 100 FTIR spectrometer, Perkin Elmer) in which the spectrometer was equipped with a liquid nitrogen cooled MCT detector and a grazing angle specular reflectance accessory (AGA, Pike Technologies). Spectra were recorded and compared for both the thermostability test and the cross-cut tape adhesion test.

### Leaching test

To validate the potential of leaching molecules from the modification layers, the modified surfaces, including bare coatings of benzoyl-PPX and maleimide-PPX, as well as the modification of immobilized CHX, BMP-2, and RGDACC, were soaked in 2 ml PBS (Sigma Aldrich) and were incubated at 37 °C for 30 days. Finally, the PBS supernatant retrieved from each sample was analyzed by using an HPLC-MS/MS system (Agilent Technologies, USA) to identify the trace of possible molecules including benzoyl and maleimide moieties, and CHX, BMP-2, and RGDACC residuals.

### Cell viability and proliferation

Mouse embryonic fibroblasts (3T3, BALA/3T3 clone A31, CCL-163) were obtained from American Type Culture Collection (ATCC). The cells were seeded at a density of 1.5 × 104 cells/cm^2^ on cell culture plates (96-well, Corning). Tissue culture polystyrene (TCPS) plates that were modified with the coatings of benzoyl-PPX and maleimide-PPX, or the subsequent modifications with CHX, BMP-2, and RGDACC, were used for the cell culture. Bare TCPS plates were served as control surfaces and were examined in parallel for the comparison. 3T3 fibroblasts were then cultured in Dulbecco’s modified Eagle’s medium (Biological Industries) containing 10% fetal bovine serum (FBS, Biological Industries) and 1% Penicillin-Streptomycin Amphotericin B Solution (P/S/A, catalog # 03-033-1B, Biological Industries), and the culturing conditions were maintained at 37 °C with 5% CO_2_ and 100% humidity. After 24 hours (or 72 hours) of the incubation, the cell viability was measured using the MTT assay. Briefly, the culture medium was removed and replaced with 200 μL of the new culture medium containing 3-[4,5-dimethylthiazol-2-yl]-2,5-diphenyl tetrazolium bromide (MTT, Sigma-Aldrich) at a final concentration of 0.5 mg/mL. The cells were then incubated for 3 hours at 37 °C to allow the formazan precipitate to form. Subsequently, the culture medium in each well was removed, and the resulting formazan was solubilized by 200 μL of 100% dimethylsulfoxide (DMSO). The quantity of formazan (directly proportional to the number of live cells) was measured by recording differences in absorbance at 570 nm using a microplate reader (ELX800, BioTek Instruments). The results were standardized according to the negative control values (% of negative control) obtained. A live/dead viability kit (Invitrogen) was used to assess the number of live/dead cells present in the cultures. After washing the cultures with sterile PBS, the supernatant was replaced with 200 μL PBS solution containing 2 μM calcein AM and 4 μM ethidium homodimer. Samples were incubated in the dark at room temperature for 30 minutes and then mounted on a microscope slide in fresh PBS. The cells were imaged using an inverted Zeiss LSM-780 confocal microscope with a 10 × objective, and the image processing software Fiji (ImageJ), was used to quantify cell number. Statistical analysis was performed with Prism 4 software (Graphpad, Inc.) using one-way ANOVA with Tukey post-test with a confidence interval of 95%. However, the murine macrophage (MΦ) cell line RAW264.7 was purchased from bioresource collection and research center (BCRC) of Taiwan, and the MΦs were cultured in Dulbecco’s Modified Eagle’s Medium containing 10% FBS and 1% P/S/A at 37 °C in a humidified atmosphere containing 5% CO_2_ and 95% air. Cells were routinely detached by mechanical force and subsequently split in a ratio of 1:5. For the examination in MTT and live/dead assays, cells were seeded at a density of 1 × 10^5^ cells/cm^2^ in 96- or 24-well of modified TCPS plates and were subsequently cultured for 24 hours and 72 hours for further characterization. Notably, a positive control surface containing MΦs that were stimulated with lipopolysaccharide (LPS, prepared in 1× PBS; final concentration 100 ng/mL) in 2 hours after cell seeding, and a negative control of bare TCPS plate, were examined in parallel for the comparison.

### Macrophage MΦ morphology

The cultured MΦs at each time point were washed twice with PBS, fixed by 4% paraformaldehyde for 15 minutes, permeabilized with 0.1% Triton X-100 (Sigma-Aldrich) for 5 minutes, and then stained with 1 µg/mL 4′,6-diamidino-2-phenylindole (DAPI, Life Technologies) and 50 µg/mL Rhodamine-Phalloidin (Life Technologies) for 15 and 30 minutes, respectively. Images were acquired using fluorescence microscopy (Nikon) and confocal laser scanning microscopy (CLSM, Leica Microsystems). Cell length and nuclear diameter of the MΦs were measured by using ImageJ. More than 20 individual cells were measured for further statistical analysis.

### Characterization of cytokines

The secretions of TNF-α and IL-1β were measured in the conditioned media from the cultured MΦs using commercially available, enzyme-linked immunosorbent assay kits (Novex^®^/Life Technologies), according to the instructions from the manufacturer. All samples were measured at 450 nm and referenced at 630 nm using a microplate reader (Instruments ELX800, BioTek). Intracellular proteins were collected by transferring samples to microcentrifuge tubes containing 1× CyQUANT cell lysis buffer (Life Technologies) and quantified by mixing the samples with a Pierce™ BCA Protein Assay Kit (Life Technologies). The values were read at 570 nm using the microplate reader. Serially diluted recombinant standards for each cytokine of interest were used to create a standard curve for determining cytokine concentrations in the conditioned media that were normalized to the amount of intracellular total protein (and expressed as pg/μg cellular protein).

### Characterization of nitric oxide

Accumulated NO concentrations in the conditioned media from the cultured MΦs were measured as nitrite by the Griess reagent (G4410, Sigma-Aldrich). Each cultured medium (100 μL, supernatant) was mixed with 100 μL of Griess reagent and incubated at room temperature for 15 minutes. The absorbance of the mixture was determined at 570 nm and referenced at 630 nm using a microplate reader (Instruments ELX800, BioTek). The nitrite levels were determined through a standard curve established with NaNO_2_.

### Statistical analyses

All data were reported as the mean ± S.D., and representative of three or more independent experiments. Differences between the experimental groups and the control groups were characterized using Student’s t-test and P-values.

## Supplementary information

Supplementary Information
