# Supplementary material for: Characterization of Mechanical Stability and Immunological Compatibility for Functionalized Modification Interfaces
Source: Sci Rep. 2019 May 21;9:7644. doi: 10.1038/s41598-019-43999-6 (PMC6529445; doi:10.1038/s41598-019-43999-6)
Supplement: Supplementary file 1 — Supplementary Information [file 41598_2019_43999_MOESM1_ESM.pdf]

Supporting Information

Characterizations of Mechanical Stability and Immunological Compatibility for Functionalized Modification Interfaces

Yao-Tsung Hsu[1], *Chih-Yu Wu*[1,]*, *Zhen-Yu Guan1*, *Ho-Yi Sun*[1], *Chieh Mei*[1,] *Wen-Chien Chen*[2], *Nai-Chen Cheng*[3], *Jiashing Yu*[1,]*, *Hsien-Yeh Chen*[1,]*

[1] Department of Chemical Engineering, National Taiwan University, Taipei 10617, Taiwan
[2] Department of Surgery, National Taiwan University Hospital, Taipei 10018, Taiwan
[3] Department of Orthopedic Surgery, Chang Gung Memorial Hospital, College of Medicine Chang Gung University, Taoyuan 333, Taiwan.

*To whom correspondence should be addressed: picorna.tw@yahoo.com.tw (C.-Y. Wu);

jiayu@ntu.edu.tw (J. Yu); hsychen@ntu.edu.tw (H.-Y. Chen)

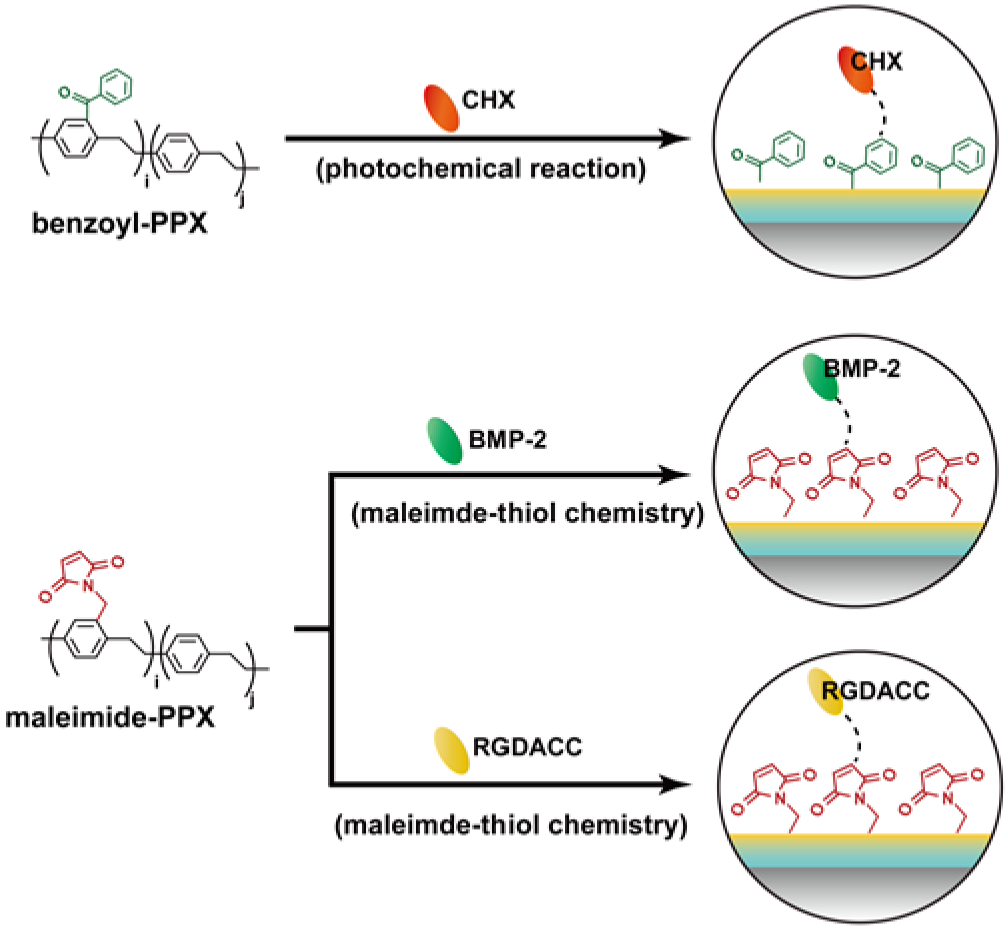

Figure S1. A summary scheme of the chemical conjugations by using the coatings in the study.

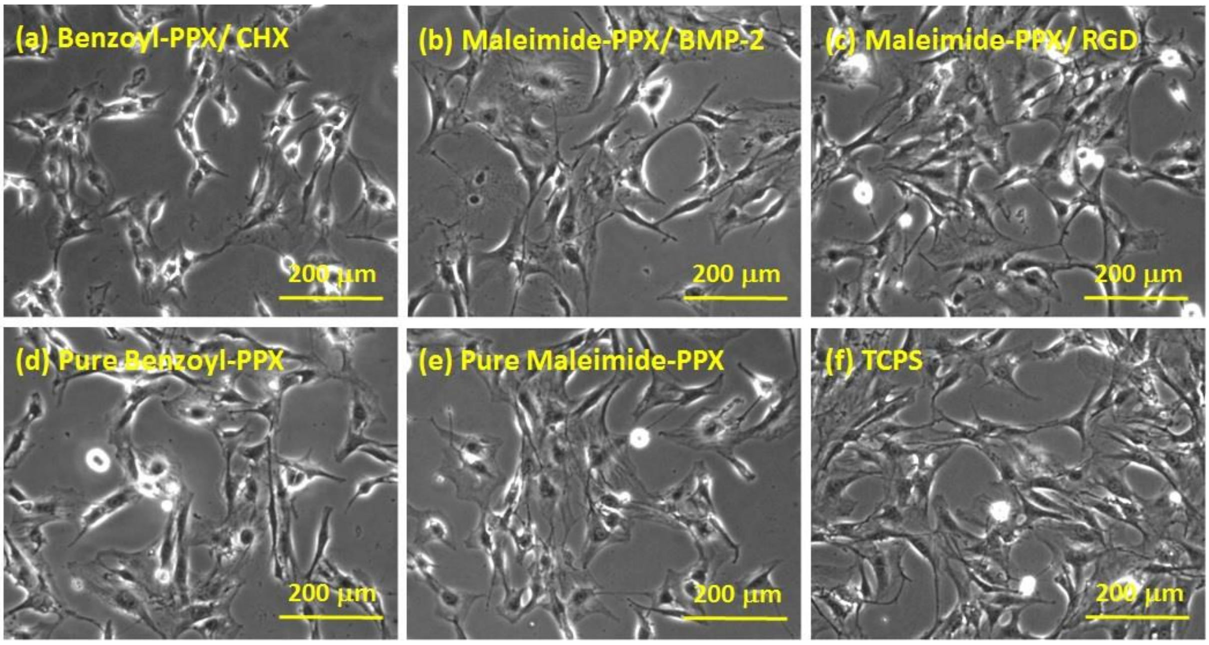

Figure S2. Optical micrographs showing the growing patterns of 3T3 fibroblasts cultured on the modified surfaces including (a) bare benzoyl-PPX, (b) bare maleimide-PPX, (c) CHX-immobilized surface, (d) BMP-2-immobilized surface, (e) RGDACC-immobilized surface, and (f) TCPS surface. Images were captured after 24 hours of cell culture.

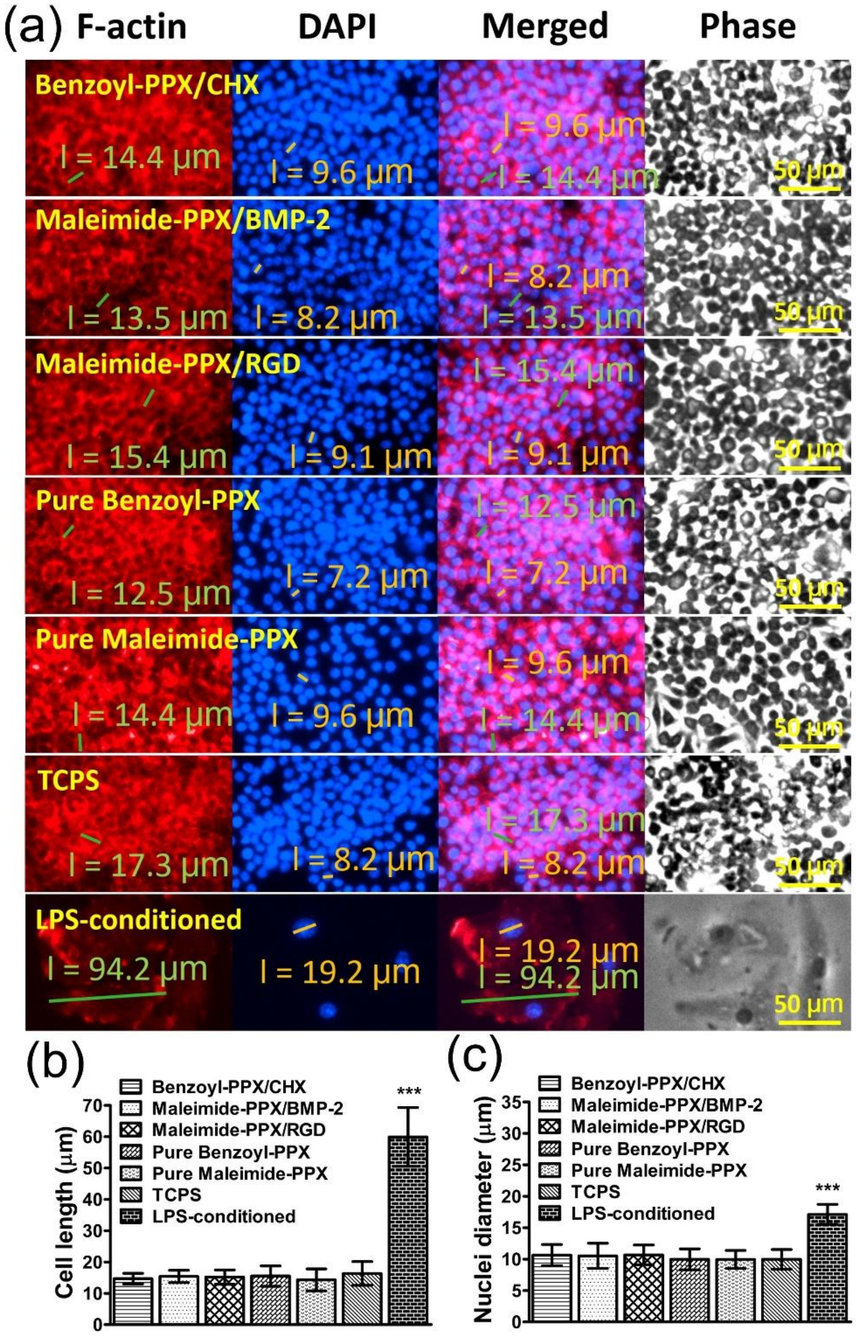

Figure S3. Analysis of the MΦ morphology on the modified surfaces after 120 hours of cell culture. (a) Compiled images including the fluorescence micrographs of the stained cytoskeleton (F-actin, red channel) and nuclei (DAPI, blue channel), and the overlaid images of both red and blue channels. Phase-contrast micrographs were also shown for the comparison. The statistical results of (b) cell length and (c) nuclei diameter of the cultured MΦs on the studied surfaces. (*** $P < 0.001$).
